# High-Accuracy Tomato Leaf Disease Image-Text Retrieval Method Utilizing LAFANet

**DOI:** 10.3390/plants13091176

**Published:** 2024-04-23

**Authors:** Jiaxin Xu, Hongliang Zhou, Yufan Hu, Yongfei Xue, Guoxiong Zhou, Liujun Li, Weisi Dai, Jinyang Li

**Affiliations:** 1 College of Computer and Information Engineering, Central South University of Forestry and Technology, Changsha 410004, China; 20215154@csuft.edu.cn (J.X.); zhou@csuft.edu.cn (H.Z.); 20213738@csuft.edu.cn (Y.H.); xueyongfei@csuft.edu.cn (Y.X.); 20212894@csuft.edu.cn (W.D.); youunique1@csuft.edu.cn (J.L.); 2 Department of Soil and Water Systems, University of Idaho, Moscow, ID 83844, USA; liujunl@uidaho.edu

**Keywords:** LAFANet, TLDITRD, LFA, FNE-ANS, AR, image-text retrieval, cross-modal

## Abstract

Tomato leaf disease control in the field of smart agriculture urgently requires attention and reinforcement. This paper proposes a method called LAFANet for image-text retrieval, which integrates image and text information for joint analysis of multimodal data, helping agricultural practitioners to provide more comprehensive and in-depth diagnostic evidence to ensure the quality and yield of tomatoes. First, we focus on six common tomato leaf disease images and text descriptions, creating a Tomato Leaf Disease Image-Text Retrieval Dataset (TLDITRD), introducing image-text retrieval into the field of tomato leaf disease retrieval. Then, utilizing ViT and BERT models, we extract detailed image features and sequences of textual features, incorporating contextual information from image-text pairs. To address errors in image-text retrieval caused by complex backgrounds, we propose Learnable Fusion Attention (LFA) to amplify the fusion of textual and image features, thereby extracting substantial semantic insights from both modalities. To delve further into the semantic connections across various modalities, we propose a False Negative Elimination-Adversarial Negative Selection (FNE-ANS) approach. This method aims to identify adversarial negative instances that specifically target false negatives within the triplet function, thereby imposing constraints on the model. To bolster the model’s capacity for generalization and precision, we propose Adversarial Regularization (AR). This approach involves incorporating adversarial perturbations during model training, thereby fortifying its resilience and adaptability to slight variations in input data. Experimental results show that, compared with existing ultramodern models, LAFANet outperformed existing models on TLDITRD dataset, with top1, top5, and top10 reaching 83.3% and 90.0%, and top1, top5, and top10 reaching 80.3%, 93.7%, and 96.3%. LAFANet offers fresh technical backing and algorithmic insights for the retrieval of tomato leaf disease through image-text correlation.

## 1. Introduction

Tomato, as a crucial agricultural crop globally, has long been the focus of attention in the agricultural sector regarding the prevention and control of leaf diseases [1]. Among these, six prominent leaf diseases include bacterial leaf spot, early blight, late blight, powdery mildew, leaf curl virus, and leaf mold. With the continuous advancement of multimodal and large-scale modeling technologies, researchers have increasingly focused on employing cross-modal techniques to address practical problems. For tomato leaf diseases, rapid access to abundant data related to tomato leaf diseases through image-text retrieval technology can facilitate precise and timely implementation of control strategies and preventive measures. This approach holds the potential to bring about innovative transformations in the field of tomato cultivation and disease management.

Traditional methods for crop disease retrieval [2] typically involve vegetable farmers inspecting diseases based on their own experience or seeking guidance from relevant technical personnel and agricultural experts. However, manual methods suffer from drawbacks such as high workload, low retrieval efficiency, inconsistent retrieval quality, and reliance on subjective judgment. In the agricultural domain, with the maturation of deep learning technology, complex single-modal management of tomato leaf diseases has been achieved [3,4,5,6,7,8,9,10,11]. Recently, with the diversification of agricultural data types [12], scholars are increasingly inclined towards employing cross-modal information retrieval techniques through various modalities, which provides valuable insights for our research. Image-text retrieval possesses the characteristic of cross-referencing multimodal data, integrating various modalities such as images and text. It has found widespread applications in fields such as healthcare, transportation, and art [13,14,15]. In the agricultural domain, crop diseases exhibit increasingly diverse modalities [16], making it challenging for single-modal image or text retrieval to meet demands. Relying solely on one type of information, whether it is image or text, exposes limitations in achieving complete understanding and intuitive interpretation of tomato leaf diseases. Thus, the limitations of using only one type of approach have fueled the swift advancement of cross-modal retrieval within the agricultural sphere [17,18].

According to the literature review, there are currently two main types of image-text retrieval methods: cross-attention strategies and visual semantic embedding techniques. The summary of image-text retrieval methods is presented in Table 1. Usually, in visual semantic embedding techniques, dual-branch neural networks are utilized to individually align text and images into a common embedding space. This facilitates faster inference and significantly enhances efficiency in real-world scenarios through embedding caching. Initially, Frome and colleagues introduced the visual semantic embedding model [19], employing CNN and Skip-Gram for feature extraction from both visual and textual inputs. Later, Faghri et al. [20] presented the VSE++ approach, enhancing it with an online hard negative selection technique within the loss function triplet, paving the way for more thorough investigations. Chen and his team [21] suggested a universal pooling technique, which dynamically decides the best pooling strategy through learning distinct weights for each dimension of ranking. Lee et al. [22] proposed a SCAN model, which selectively integrates image regions and textual elements to gauge the similarity between images and text. Following this, Wei et al. [12] introduced a method incorporating both cross-attention and self-attention mechanisms to effectively capture interactions within and across modalities. Qu and his team [23] proposed a dynamic routing mechanism for model embedding, allowing the dynamic selection of embedding paths according to input. Additionally, four distinct types of interaction units were conceptualized by them to amplify communication across various modalities and facilitate intra- and inter-modality exchanges. Zhang and colleagues [24] pioneered the NAAF methodology, which extends the SCAN methodology, aiming to refine similarity computation by mitigating the drawbacks associated with mismatched fragments. In scientific research, false negatives are a common challenge. Li and his team [25] introduced a novel approach called False Negative Elimination (FNE) to address this issue by employing sampling techniques for negative instances. Additionally, they developed a memory module with momentum, enabling the maintenance of a substantial negative buffer and the implementation of negative sampling strategies on this buffer, ultimately leading to state-of-the-art retrieval performance.

**Figure 1:**
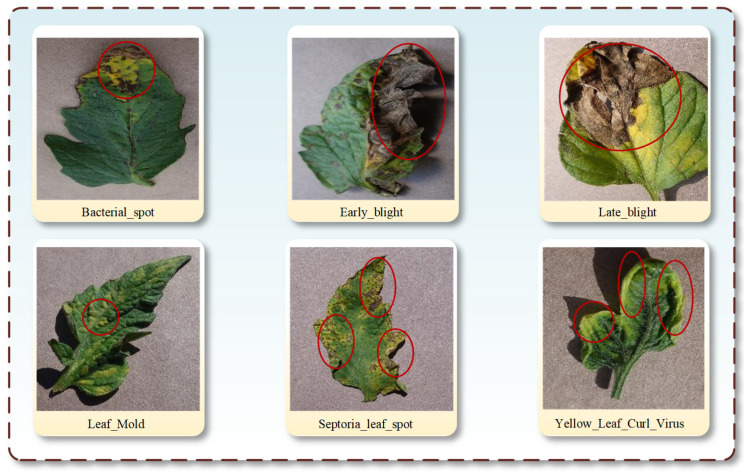
Examples of various challenges in retrieving images and text related to diseases.

During our experimental process, cross-modal retrieval of tomato leaf diseases mainly encountered three issues (as illustrated in Figure 1). 1. Background interference: when leaf diseases occur near the edges of leaves, interference from soil backgrounds increases the complexity of retrieving infected areas. Such issues exacerbate the difficulty for the model of the primary retrieval of leaf disease categories. 2. Intra-class variability: the presence of intra-class variability poses a series of serious problems for disease detection, making the presentation of the same disease more complex, including changes in color, texture, and shape. Such variations increase the complexity of model retrieval of image and text features, and intra-class variability can easily lead to a decline in model performance in real-world applications. 3. Inter-class similarity: different types of tomato leaf diseases exhibit certain similarities in external features, which may introduce some interference into our model’s accurate image-text retrieval. Inter-class similarity is often manifested as similarities in color, shape, and pathological features. Figure 1 reflects that even different diseases may exhibit similar discoloration and spots. Effectively mitigating inter-class similarity is a challenging problem that the model should deeply consider and address.

From Figure 1, it can be observed that when analyzing the similarities and differences of tomato leaf diseases in depth, we can carefully examine their commonalities and differences in color, texture, and shape, and understand why these features are difficult to accurately distinguish. For example, both early blight and late blight exhibit a deep brown color, and their spots are typically approximately circular, resulting in visual similarities that make it difficult for the human eye to differentiate between these two diseases rapidly and accurately without specialized knowledge. Similarly, leaf spot and bacterial leaf spot both display brown spots with relatively regular shapes. These similarities pose challenges for professionals and farmers in practical agricultural work, especially in large-scale tomato plantations. Therefore, learning complex patterns from many image and text data using cross-modal models can enhance the precise cross-modal retrieval capability for these diseases. Moreover, tomato plants grow in natural environments where their leaves may be surrounded by rich and complex backgrounds, including soil, other plants, and varying lighting conditions. These complex background elements may exhibit textures or colors like those of the diseased areas, making it difficult for the model to accurately separate the disease from the background. Improving methods to extract image characteristics and reduce the influence of irrelevant surroundings is vital for achieving more precise retrieval of desired objects, thereby minimizing retrieval errors.

In this study, we achieved the first ever implementation of cross-modal retrieval in the field of tomato leaf diseases. To facilitate the retrieval of information across different modes pertaining to tomato foliage issues we introduced LAFANet, leveraging advanced visual semantic embedding techniques like FNE. Addressing background interference, intra-class variability, and inter-class similarity issues in cross-modal retrieval of tomato leaf diseases, we innovatively introduced technologies such as LFA, FNE-ANS, and AR to enhance LAFANet. Our objective is to create a streamlined system for retrieving images and text related to tomato leaf issues, overcoming the constraints of relying solely on one type of retrieval method. LAFANet aims to offer reliable assistance for real-world implementations in agriculture and enhance the effective control and management of plant leaf ailments.

## 2. Method

### 2.1. The Cross-Modal Tomato Leaf Disease Retrieval Dataset

#### 2.1.1. Initial Dataset Selection and Preprocessing

The PlantVillage dataset [26] serves as the foundation of this study. We selected 6000 tomato leaf images from this dataset, encompassing six types of leaf diseases: bacterial leaf spot, early blight, late blight, powdery mildew, leaf curl virus, and leaf mold. Each category of tomato leaf disease images comprises 1000 images. This selection covers a variety of common tomato leaf diseases, allowing the model to comprehensively understand the characteristics of each disease. By providing an ample number of training samples for deep learning models, this dataset enables the model to effectively learn and differentiate between diverse types of diseases, thus enhancing its generalization capability. Sourced from actual tomato plantations, the dataset ensures the authenticity and representativeness of the images, thereby ensuring the model’s easy application in real-world scenarios.

To maintain consistency across the dataset, all images were adjusted to a size of 250 × 250 pixels. Following this, the dataset was randomly partitioned into training, validation, and testing sets, distributed in proportions of 80%, 10%, and 10%, respectively.

#### 2.1.2. Annotation and Creation of Text Data

**Figure 2:**
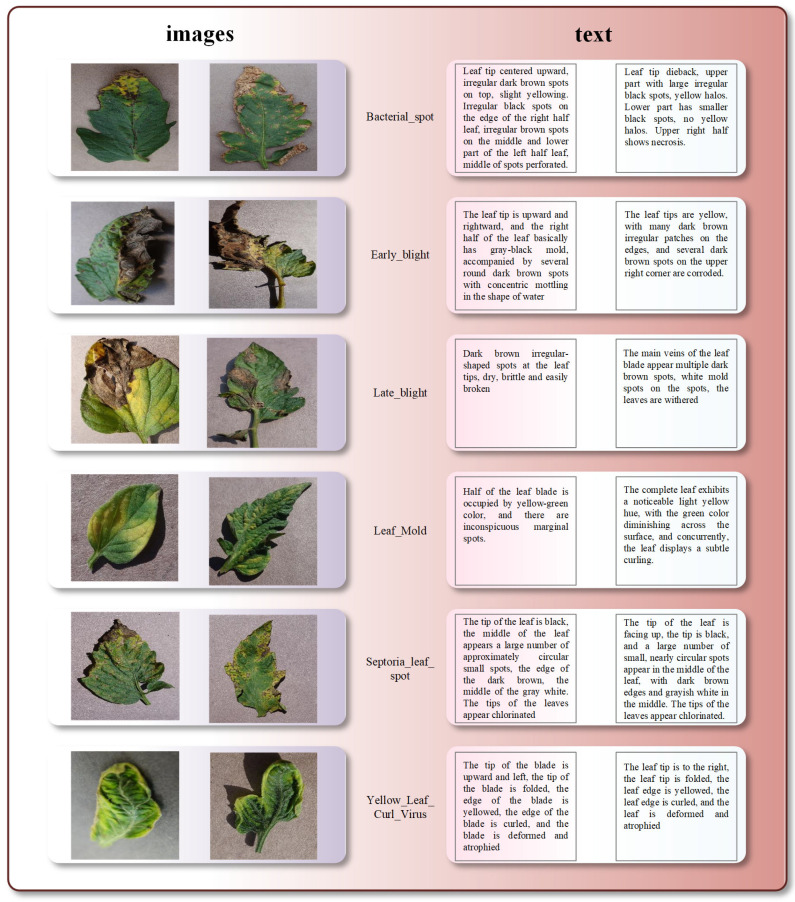
Examples of image-text pairs in the tomato leaf cross-modal retrieval dataset.

We enlisted an expert team from the Agricultural Science Institute of Changsha, Hunan Province, China, who dedicated one hundred days to meticulously describing our image dataset, resulting in 6000 images. For each type of tomato leaf disease we selected 1000 images, allowing for a uniform analysis of the data for each disease category. Thorough annotations were provided for six categories of visual images depicting tomato leaf diseases, elucidating the characteristics of the affected foliage, such as patterns, colors, locations of lesions, leaf positioning, quantity, and various semantic details. The meticulousness of these annotations aimed at ensuring fine-grained correspondence between our image and text features. Our principle in text annotation was to adhere to a unified standard for the six disease categories, with specific annotations made based on the specific characteristics of the leaves (patterns, colors, lesion locations, leaf positioning, quantity, and various semantic details). This approach enables our model to establish a robust association between images and text. These descriptions adeptly convey the semantic essence encapsulated within the visual images. Through these annotation efforts, we achieved a close association between image and text semantics. This dataset, containing corresponding image-text pairs, will be utilized to train our cross-modal image-text retrieval model for task of tomato leaf disease retrieval. Figure 2 presents examples of annotations corresponding to the six disease images. In Figure 2, bacterial spot disease primarily affects the edges, presenting gray-brown spots surrounded by yellow halos. As the disease progresses, the spots may merge into larger necrotic areas. Leaves infected with early blight exhibit small, deep brown subcircular spots, often connecting to form irregular edges and water-soaked lesions inside. Infected leaf surfaces may also exhibit small mold spots. Late blight manifests as extensive, deep brown, moist spots, with the possibility of small mold spots on the leaf underside. Differentiating between early and late blight primarily relies on the spread of spots and the distribution of mold. Leaves affected by powdery mildew display irregular yellow-green mold, often covering large areas and nearly filling the entire leaf, resulting in symptoms of bending and twisting. As its name suggests, leaf mold features gray-brown necrotic spots with yellow halos, typically larger in scale and resembling fish eyes. The last type, yellow leaf curl disease, is relatively easy to identify, with affected leaves appearing abnormally yellow overall and exhibiting wavy or serrated edges, contrasting sharply with the normal green smooth leaves. The leaves may also display bending and wrinkling, leading to stunted growth compared to ordinary leaves.

There are similarities among these six tomato leaf diseases, such as early blight and late blight having similar textures, with spots typically brown and nearly circular. However, certain diseases show multiple characteristics, such as Septoria leaf spot, which has a brown core surrounded by a yellow halo. Another example is bacterial spot, which is more prone to confusion with similar backgrounds due to the objects present at its edges. Detailed textual descriptions help in understanding the content of images and ensure reliable training data for our models. Our objective is to foster correlations between textual content and visual representations by curating a dataset comprising pairs of images and corresponding text. Through training models on this dataset, we endeavor to enhance capabilities in tasks related to retrieving text–image associations.

### 2.2. Model Framework

**Figure 3:**
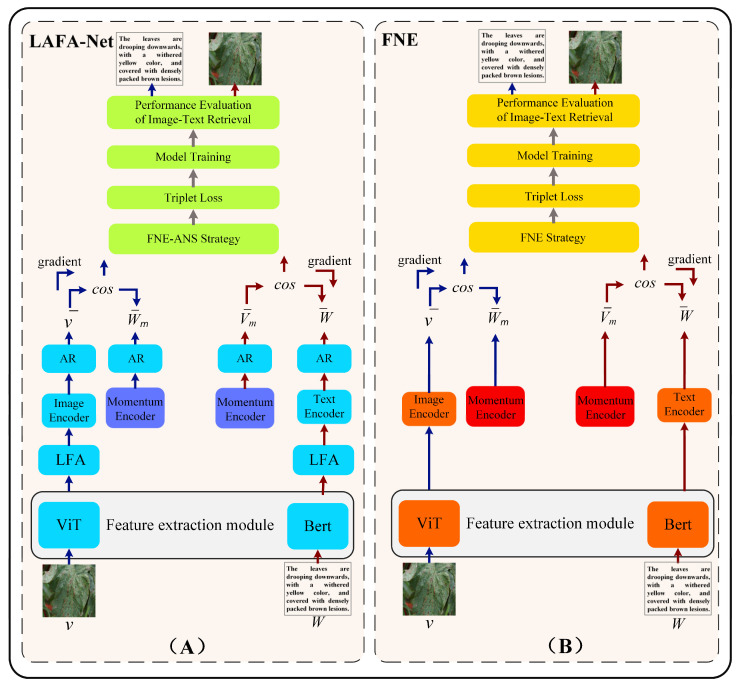
Displaying the schematic representation of our novel LAFANet model framework, alongside its baseline model FNE. (**A**) LAFA-Net; (**B**) FNE.

In Figure 3, the schematic portrayal of the LAFANet framework we propose elucidates the process of image-text retrieval for textual descriptions corresponding to images depicting tomato leaf diseases. In the depicted initial stage, features are extracted from text and image feature sequences employing specialized feature extraction modules, namely ViT and BERT. The first phase entails enhancing the attributes of image and text sequences via LFA. Following this, the derived feature sequences and their associated momentum memory units (Momentum Encoder) will be inputted into both the Image Encoder and Text Encoder. Throughout the training phase, the innovative FNE-ANS technique customizes triplet loss function to guide learning trajectory. Subsequently, the proposed AR is applied to perturb the features stored in the feature encoders, enhancing the specificity of features and the generalization of the model. Throughout the training process there arises the proposition of FNE-ANS, aiming to apply restrictions to the triplet loss function. FNE-ANS selects comprehensive negative samples to ensure the adversarial nature of the selected negative samples while cutting false negatives. The following sections will provide detailed analyses for each module. Our innovative components work together synergistically to enhance the efficacy and resilience of the cross-modal retrieval system for tomato leaf ailments, yielding remarkable outcomes.

#### 2.2.1. Feature Extraction Component

We utilized the ViT model to capture image attributes, while simultaneously utilizing the BERT model to extract textual traits. After completing feature extraction from both images and text, we employed average pooling operations to derive comprehensive features for text and image. This helps in perfecting later loss functions and calculating final similarity efficiently. This step aims to reduce data dimensionality while keeping crucial semantic information to provide richer input information for later tasks.

We chose the ViT model, built on Transformer architecture [27], for image feature extraction. Its utilization is depicted in Figure 3A. In the ViT model, the image is initially divided into smaller patches. A multi-head self-attention mechanism is then employed to effectively capture spatial context information within the image, resulting in features being extracted for these individual patches. To ensure semantic alignment between visual and textual data, we follow a similar strategy as in prior research [28,29]; the patch characteristics are aligned onto a unified semantic framework through fully connected layers. As a result, the identified attributes for each patch may be depicted as *V* = {$v_{i}/$*i =* 1, *...*, *m*, $v_{i}$ ∈ *Rd*}, where m denotes the quantity of patches per image. The formula for the image representation is shown in Equation (1):

(1) $$\;\overset{-}{v}=\frac{1}{m}\sum_{i=1}^{m}v_{i}$$

In choosing the text feature extraction module, we opted for a pre-trained BERT model [30] to ensure efficient handling of input text, aligning with current advancements in Natural Language Processing (NLP). Figure 3A shows how BERT is utilized. BERT was utilized to derive contextual word embeddings. Additionally, to align the extracted word attributes into a cohesive semantic space, fully connected layers were employed, yielding a structured representation denoted as *W* = {$w_{j}$*|j =* 1, *...*, *l*, $w_{j}$ ∈ *Rd*}. In this context, l stands for the sentence’s word count, while d signifies the feature dimensionality. This procedure aids in grasping semantic representations of textual data, thereby furnishing our model with abundant textual features. Equation (2) illustrates the text representation.

(2) $$\overset{-}{w\;}=\frac{1}{l}\sum_{j=1}^{l}w_{j}$$

#### 2.2.2. LFA

Our proposed LFA aims to enhance self-attention for both tomato leaf disease images and textual feature sequences. Below, we provide a detailed description of its unique structure. The core concept of the LFA module involves integrating learnable fusion weights, enabling the model to perform self-attention across different directions and dimensions, thereby enhancing its ability to focus on various aspects of the input data with greater flexibility. This element includes two sets of adjustable parameters, one for left self-attention and the other for right self-attention. These dual sets of weights enable the model to selectively prioritize details from both ends of the input information, facilitating a broader feature representation. Finally, these two sets of weights are fused through learnable parameters to obtain the comprehensive weights we need.

**Figure 4:**
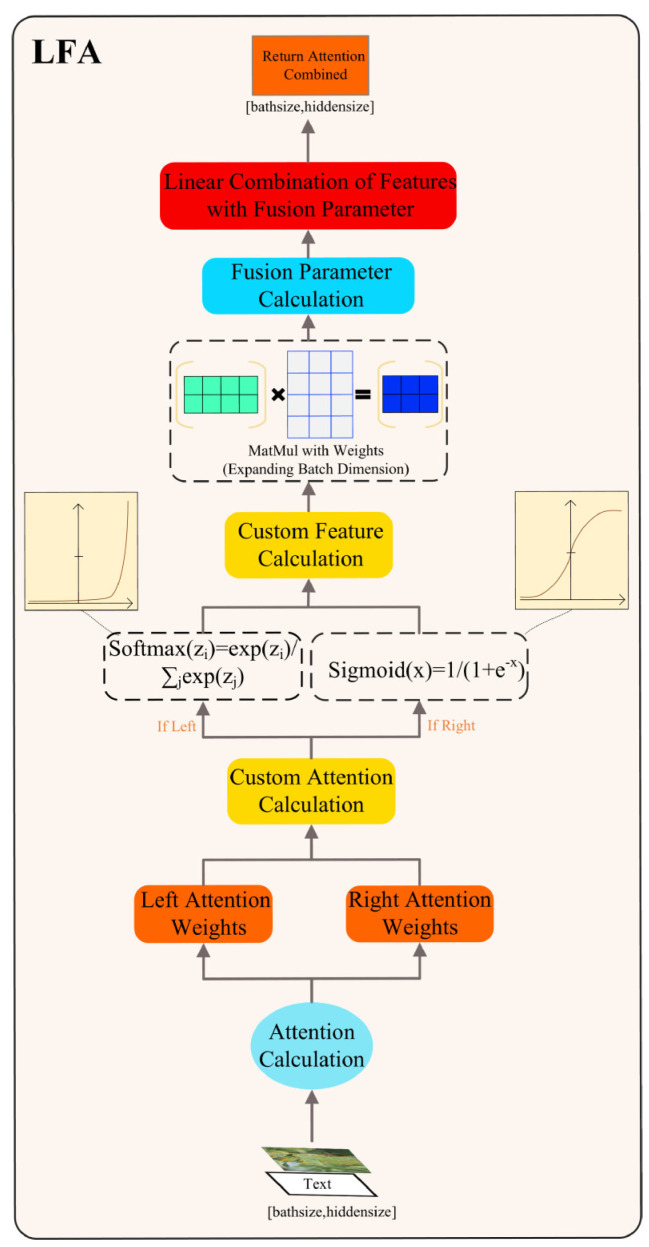
The schematic diagram of our proposed LFA.

In Figure 4 in the LFA, firstly, we establish left and right self-attention by introducing two sets of learnable weights, namely *left_attention_weights* and *right_attention_weights*. The initial weights for these two sets are established through the Xavier/Glorot initialization approach to ensure optimal initial weights. This provides flexibility to the model, enabling it to effectively learn input information in different directions of attention. Next, during the forward propagation process, we select different forward propagation methods based on whether attention masks are provided or not. If a mask is provided, we use encoder (*input_ids = inputs_adv*, *attention_mask = attention_mask*) for forward propagation, otherwise, we use encoder (*pixel_values = inputs_adv*) for forward propagation. This differential forward propagation approach ensures that the model appropriately focuses on input data in different directions.

The equations delineating the computation of left attention weights and right attention weights in LFA are expressed in Equation (3):

(3) $${\mathrm{left}}_{\mathrm{weights}}={\mathrm{custom}}_{{\mathrm{attention}}_{\mathrm{calculation}}}\;(\mathrm{left}\_\mathrm{attention}\_\mathrm{weights},\;\mathrm{hidden}\_\mathrm{size},\;\mathrm{direction}=‘\mathrm{left}’)\;{\mathrm{right}}_{\mathrm{weights}}={\mathrm{custom}}_{{\mathrm{attention}}_{\mathrm{calculation}}}\;(\mathrm{right}\_\mathrm{attention}\_\mathrm{weights},\;\mathrm{hidden}\_\mathrm{size},\;\mathrm{direction}=‘\mathrm{right}’)$$

The left self-attention weights are the degree to which the model focuses on various positions in the input sequence in the forward direction. These weights are initialized using Xavier/Glorot initialization and are used to compute attention in the forward direction. Similarly, the right self-attention weights are the degree to which the model focuses on various positions in the input sequence in the backward direction. Also initialized using Xavier/Glorot initialization, they are used to compute attention in the backward direction.

The computation procedure for obtaining the left characteristics and right characteristics in LFA is outlined in Equation (4):

(4) left_attended_features=custom_feature_calculation(left_weights, X, direction=‘left’)right_attended_features=custom_feature_calculation(right_weights, X, direction=‘right’)

The ${\mathrm{left}}_{{\mathrm{attended}}_{\mathrm{features}}}$ are obtained by weighting and summing the input sequence using the left self-attention weights. This process is implemented through matrix multiplication, highlighting the crucial features in the left direction. Similarly, the ${\mathrm{right}}_{{\mathrm{attended}}_{\mathrm{features}}}$ are obtained by weighting and summing the input sequence using the right self-attention weights. Also implemented through matrix multiplication, this emphasizes the noteworthy features in the right direction.

LFA introduces a learnable fusion parameter, ${\mathrm{fusion}}_{\mathrm{parameter}}$, which is used to weight and fuse the left and right features. The attention fusion process is described by Equation (5):

(5) $${\mathrm{attention}}_{\mathrm{combined}}={\mathrm{fusion}}_{\mathrm{parameter}}\;\times \;{\mathrm{left}}_{{\mathrm{attended}}_{\mathrm{features}}}\;+(1-{\mathrm{fusion}}_{\mathrm{parameter}})\;\times {\;\mathrm{right}}_{{\mathrm{attended}}_{\mathrm{features}}}$$

It serves the purpose of modifying the fusion ratio between left and right features, empowering the model to dynamically determine the influence of left and right features on the ultimate representation, guided by the data. This mechanism enhances the model’s comprehensive use of information from different directions, thereby improving the diversity of representations.

Algorithm 1 presents the pseudocode, detailing enhancements to image and text sequence features via the LFA module.

LFA boosts the extraction of vital semantic details by integrating heightened directional focus into the representations of text and images. Incorporating self-attention mechanisms across various orientations, it grants the model increased adaptability, empowering it to grasp and fuse information from images and text more thoroughly. This enhances the model’s sensitivity to semantic information and its ability to capture it. Such a design makes LFA perform superiorly in addressing the complexities of real-world image-text matching problems.
**Algorithm 1:** LFA Algorithm**Input:** Order of Image or Text Characteristics **Output:** Sequence of Image or Text Features Enhanced through LFA Mechanism Pseudocode: class LFA:    def __init__ (self, input_size, num_heads = 32):        super (LFA, self). __init__ ()         self.num_heads = num_heads        self.left_attention_weights = nn.Parameter (nn.init.xavier_uniform_ (torch.empty(input_size, input_size)))        self.right_attention_weights = nn.Parameter (nn.init.xavier_uniform_ (torch.empty(input_size, input_size)))        self.fusion_parameter = nn.Parameter (torch.randn(1))    def forward (self, x):        batch_size, hidden_size = x.size()        left_weights = self.left_attention_weights.unsqueeze(0).expand (batch_size, −1, −1)        right_weights = self.right_attention_weights.unsqueeze(0).expand (batch_size, −1, −1)        left_weights = self.custom_attention_calculation (left_weights, hidden_size, direction = ‘left’)        right_weights = self.custom_attention_calculation (right_weights, hidden_size, direction = ‘right’)        left_attended_features = self.custom_feature_calculation (left_weights, x, direction = ‘left’)        right_attended_features = self.custom_feature_calculation (right_weights, x, direction = ‘right’)        attention_combined = self.fusion_parameter * left_attended_features + (1 − self.fusion_parameter) * right_attended_features        return attention_combined    def custom_attention_calculation (self, weights, hidden_size, direction):        if direction == ‘left’:            weights = F.softmax (weights/hidden_size*0.3, dim = −1)        elif direction == ‘right’:            weights = F.sigmoid (weights)        return weights    def custom_feature_calculation (self, weights, x, direction):        if direction == ‘left’:            attended_features = torch.matmul (weights, x.unsqueeze(2)).squeeze(2)        elif direction == ‘right’:            attended_features = torch.matmul (weights, x.unsqueeze(2)).squeeze(2)        return attended_features


#### 2.2.3. FNE-ANS

**Figure 5:**
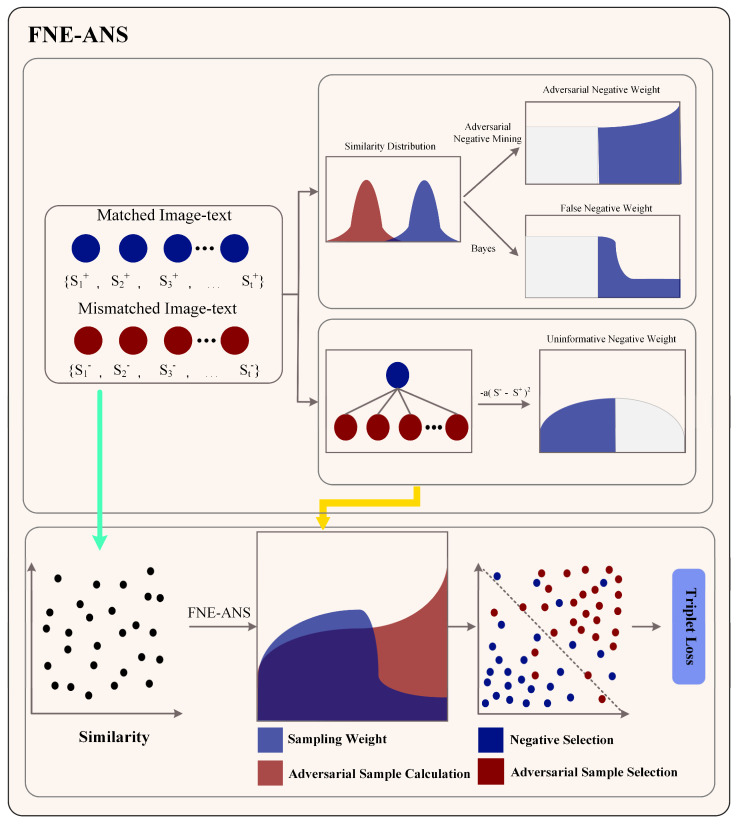
The schematic diagram of our proposed FNE-ANS.

In this section, we will delve into the fundamental principles of the FNE-ANS strategy and provide a detailed explanation of the two constituent methods of the proposed strategy: FNE and ANS. Figure 5 illustrates the core principles of the FNE-ANS approach. We employ FNE to reduce false negatives in tomato leaf disease samples, addressing the inter-class similarity challenges in cross-modal image-text retrieval, while ANS accelerates model training speed and enhances performance. ANS identifies the most severe negative instances associated with tomato leaf diseases, thereby alleviating issues of disease similarity across different modalities in tomato image-text retrieval.

By merging the FNE strategy with the ANS strategy in a weighted manner, we amalgamate these pivotal strategies to enhance the selection of negative samples tailored for anchor points, thereby introducing the novel FNE-ANS strategy. The incorporation of FNE-ANS is geared towards maximizing the advantages derived from both methodologies and bolstering the equilibrium of their influences. By employing the FNE strategy, FNE-ANS mitigates the model’s inclination to erroneously disregard negative samples aligned with the anchor points. The first step in the FNE methodology is to assess the probability that negative sample is mistakenly labeled as a false negative. After executing this method, we assign reduced weights to negative samples showing heightened probabilities of being false negatives by employing an exponential activation function for weight distribution. Concurrently, the FNE-ANS technique assists the model in enhancing its ability to differentiate between similar image-text pairs by implementing the ANS strategy, thereby identifying and prioritizing the most formidable negative samples for training. When proposing the ANS strategy, we consider both the complexity of the samples and their respective significance.

**FNE:** after buying the comprehensive features of tomato leaf disease images and text as shown in Figure 5, the FNE approach utilizes triplet loss to align visual and textual meanings. Initially, the evaluation function is employed to assess the proximity or similarity between the vector representation of the image $\bar{v}$ and that of the sentence $\bar{w}$, thereby determining whether they depict affirmative or adverse cases. We uphold the traditional interpretation of cosine similarity in this context.

(6) $$s(\bar{v},\bar{w})=\frac{{\bar{v}}^{T}\bar{w}}{\left\|\bar{v}\|\|\bar{w}\right\|}$$

By employing the triplet dissimilarity metric, our objective is to cluster comparable instances while distancing dissimilar ones. Here follows a succinct overview:

(7) $$L_{\mathrm{tri}\;}\;={\left[\mathrm{margin}-s(\bar{v},\bar{w})+s\left(\bar{v},{\bar{w}}^{-}\right)\right]}_{+}\;+{\left[\mathrm{margin}-s(\bar{v},\bar{w})+s\left({\bar{v}}^{-},\bar{w}\right)\right]}_{+},$$

Designed to ensure a substantial difference in distance between negative samples and anchor points, compared to positive samples, the margin serves as a constraint hyperparameter. This shows a restriction on the relatively distant position of negative samples compared to positive samples in the feature space; otherwise, the triplet will be penalized. We use [·] + = max (0, ·) to denote this constraint. In a mini batch, ${\bar{w}}^{-}$ represents the textual embedding of challenging negative instances, whereas ${\bar{v}}^{-}$ signifies the image embedding of difficult negative instances.

Usually, conventional methods for mining hard negative samples tend to pick samples that exhibit the greatest resemblance to the anchor point, thereby establishing triplets of <anchor, positive sample, negative sample>. Yet, as previously noted, challenging negative samples exhibiting remarkably high similarity might align semantically with the anchor point. Since this alignment occurs owing to the wide array of semantic variations, we refer to them as false negatives in this paper. This means that they are labeled as negative samples but match the anchor point. This could lead to confusion during training, thereby affecting the performance of image-text matching. In this situation, FNE effectively tackles this problem. The core idea behind the FNE strategy revolves around reducing false negatives through the reduction of the probability for negative samples to be wrongly classified as such. This approach employs Bayesian principles, employing prior probability distributions for positive and negative samples to evaluate the probability of false negatives among negative samples. In this manner, the FNE strategy looks to lower the likelihood of false negatives, thus addressing the concern. In short, the FNE approach tries to gauge the chance of an adverse instance being mistakenly marked as a false negative. It does this by using Bayesian principles and considering the initial likelihoods of positive and adverse instances in each instance.

Instances labeled as negative within the dataset, yet conceptually corresponding to the reference point, are denoted as false negatives, as previously illustrated. When employing posterior probabilities to assess the chance of negative instances being misclassified as false negatives, we are gauging their probability of conforming to the reference point or turning into positive samples. Therefore, it is crucial to examine the statistical attributes of affirmative cases utilizing correlation scores, which depict the resemblance between the anchor and its respective positive example. Moreover, it is essential to emphasize the significance of optimizing the distribution of negative instances, particularly when factoring in the potential occurrence of false negatives within negative samples. In summary, we calculate the similarity between positive and negative samples as follows:

(8) $$S^{+}\;=\left[s_{1}^{+},s_{2}^{+},s_{3}^{+},\cdots ,s_{i}^{+},\cdots \right]S^{-}\;=\left[s_{1}^{-},s_{2}^{-},s_{3}^{-},\cdots ,s_{i}^{-},\cdots \right],$$

We denote sets $S^{+}$ and $S^{-}$ to be the similarity of matched and mismatched pairs, respectively. We model similarity using a normal distribution, depicted as follows:

(9) $$\begin{array}{l} f_{S|c}(s)=\frac{1}{\sigma^{+}\sqrt{2\pi }}e^{\left[-\frac{{\left(s-\mu^{+}\right)}^{2}}{2{\left(\sigma^{+}\right)}^{2}}\right]}\\ f_{S|\bar{c}}(s)=\frac{1}{\sigma^{-}\sqrt{2\pi }}e^{\left[-\frac{{\left(s-\mu^{-}\right)}^{2}}{2{\left(\sigma^{-}\right)}^{2}}\right]} \end{array}$$

Here, (*μ+*, *σ+*) and (*μ−*, *σ−*) signify the centroid and variability of the two distributions, which are progressively aggregated and calculated within each mini-batch. *c* and *c*^−^ denote stochastic occurrences for concordance and discordance, respectively, showing whether a pair is matched or mismatched. It is crucial to emphasize our avoidance of pre-existing models or offline characteristics in shaping these distributions. As training proceeds, the model’s representational capability steadily enhances. In simpler terms, fixed distributions are not precise enough to capture the statistical characteristics of similarity, thus they cannot effectively eliminate false negatives in the current model. Considering that the current model surpasses the capabilities of its predecessor, the latter falls short in offering sufficiently challenging instances and criteria to identify false negatives, thus hindering the progress of the current model. However, given the inherent margin of error in the similarity calculations of the current model, it is crucial to attain a more accurate assessment of the genuine similarity distributions for both matching and non-matching pairs. To achieve this, similarity is only sampled when the likeness of matched pairs within the current mini batch exceeds that of other dissimilar pairs. This procedure aids in deciding on the central tendency and dispersion for constructing the probability density function.

Once we have the first similarity distribution, we can decide on the probability of an adverse specimen becoming an incorrect negative classification. The likelihood of this occurrence is represented as *P*(*C = c*∣*S = s*), where *c* signifies the sample matching the anchor point in significance, and *s* signifies the degree of resemblance between them. However, because *C* is categorical and *S* is continuous, computing *P*(*C = c*∣*S = s*) directly is not viable due to the impossibility of *S = s* having a non-zero probability for a continuous variable. Following the methodology FNE, we define the condition as *s ≤ S ≤ s +* Δ*s*, with Δ*s* representing a minute positive increment, and subsequently converging as Δ*s* approaches zero. By applying Bayes’ theorem, the probability of class *C* being *c*, given the observed evidence *S* being *s*, is determined as follows:

(10) $$\begin{array}{c} P(C=c|S=s)\approx P(C=c|s\leq S\leq s+\Delta s)\\ \;\;\;\;\;\;\;\;\;\;\;\;\;\;\;\;\;\;\;\;\;\;\;=\frac{P(c)P(s\leq S\leq s+\Delta s|c)}{P(s\leq S\leq s+\Delta s)} \end{array}$$

Our next step involves utilizing the principles of the mean value theorem in integral calculus, where s represents the likeness between the anchor and the sample.

(11) $$\begin{array}{ll} \frac{P(c)P(s\leq S\leq s+\Delta s|c)}{P(s\leq S\leq s+\Delta s)}= & \frac{P(c)\int_{s}^{s+\Delta s}f_{S|c}(t)dt}{\int_{s}^{s+\Delta s}f_{S}(t)dt}\\ & \approx \frac{P(c)f_{S|c}(s)\Delta s}{f_{S}(s)\Delta s}\\ & =\frac{P(c)f_{S|c}(s)}{f_{S}(s)} \end{array}$$

Following the principle of total probability as prescribed by the law, denominator $f_{S}(s)$ can be deduced via the subsequent computation.

(12) $$f_{S}(s)=P(c)f_{S|c}(s)+P(\bar{c})f_{S|\bar{c}}(s),$$

The occurrence wherein the sample deviates from the anchor point is denoted by $\bar{c}$. Ultimately, we can reformulate Equation (13) as follows:

(13) $$P(C=c|S=s)=\frac{P(c)f_{S|c}(s)}{P(c)f_{S|c}(s)+P(\bar{c})f_{S|\bar{c}}(s)}$$

The division of the computation for posterior probability *P*(*C* = *c*∣*S* = *s*) into 3 channels is depicted in the previous derivation. The primary pair of distributions, being the resemblance between matched and mismatched pairs, have already been derived in Equation (14). Given the presence of both matching and mismatching instances for a specified specimen and anchor entity, the stochastic variable *C* adheres to a Bernoulli distribution. Thus, we estimate the posterior probability by evaluating the following two similarity distributions:

(14) C~B(1, P),

The probability of event C is represented by P. As false negatives are present within the dataset, directly computing the parameter of the distribution P from annotated data is inappropriate. To tackle this problem, we adjust P and decide on the best value through validation set choice.

For negative samples concerning the anchor point, we can obtain their probabilities of being false negatives from Equation (13). Afterward, it is imperative to ascertain whether to refine the negative samples within triplet loss by utilizing the probabilities derived from false negatives. Despite its apparent simplicity, the threshold method conceals its complexity, demanding careful tuning of the hyperparameter in the form of the threshold probability. Opting for a strategy that is more instinctual, we encounter negative samples within triplets with a reduced frequency of being false negatives as a result. Achieving this entails the utilization of weighted sampling, which hinges on posterior probabilities. Opting for an exponential activation function, we aim to enhance the smoothness of the sampling process. This succinctly outlines entire procedure:

(15) $$\mathrm{pi}\;=\exp\left(-P\left(C=\;c|S\;=\;s\left(d_{i}^{-}\right)\right)\right),$$

The term *pi* used to denote the weight assigned to the selection of negative samples in this context is referred to as the sampling coefficient, whereas these negative samples are symbolized by $d_{i}^{-}$. Using the sampling method mentioned earlier helps to reduce the chance of encountering false negatives. However, we suggest employing a reduction approach to address less complex negative scenarios and direct optimization efforts accordingly. These instances, with a minimal likelihood of being erroneous negatives, are unsuitable for the FNE sampling method. This is because these negative samples are clearly disparate from the designated anchor point and will never correspond to it. As elucidated in [31,32,33], the incorporation of these simplistic negative samples within triplets fails to furnish the crucial insights necessary for augmenting model efficacy. Consequently, for negative samples exhibiting near-zero probabilities of false negatives, we diminish their sampling weights and modify them as follows:

(16) $$\mathrm{pi}=\exp(-a(s\left(s\left(d_{i}^{-}\right)-s\left(d^{+}\right){)}^{2}\right),$$

in this scenario, the parameter “a” serves the purpose of regulating sampling density, operating at a level of depth adjustment. In summary, FNE incorporates these two methodologies through the utilization of negative sampling in the following fashion:

(17) $$p_{i}=\left\{\begin{array}{ll} \exp\left(-P\left(C=c|S=s\left(d_{i}^{-}\right)\right)\right), & \mathrm{others}\;\\ \exp\left(-\alpha {\left(s\left(d_{i}^{-}\right)-s\left(d^{+}\right)\right)}^{2}\right), & P\left(C=c|S=s\left(d_{i}^{-}\right)\right)\leq \lambda^{2} \end{array}\right.$$

λ is scheduled to 0.01. This guarantees that incorrect negative determinations and effortlessly identifiable negative samples are assigned lower sampling weights, aiding the model in learning semantic portrayals from authentic and difficult negative samples.

Using the FNE strategy enables us to reduce false negatives in tomato leaf disease samples, addressing inter-class similarity challenges in cross-modal image-text retrieval for tomatoes.

**ANS Strategy:** Figure 5 depicts the utilization of the ANS approach in enhancing triplet loss by identifying the most formidable negative instances, thereby expediting training and enhancing model efficacy. The procedural outline of the ANS approach in Figure 5 is delineated below.

Step 1: calculate the matching degree metric. The process of calculation entails employing the matrix dot product *@*, where the matrix *im* representing image embeddings and the matrix *s* representing text embeddings are subjects of computation to determine their alignment. The specific formula is given by Equation (18).

(18) scores=im@ s.T

Step 2 involves the derivation of diagonal scores. Following this, the diagonal elements of the similarity score matrix are computed, and variables d1 and d2 are subsequently assigned. When compared with itself, these numerical values depict the likeness scores between text or image. The specific formula is given by Equation (19).

(19) $$\begin{array}{c} diagonal=scores.diag().view\left(im.size\left(0\right),1\right)\\ d1=diagonal.expand\_as(scores)\\ \;\;\;\;d2=diagonal.t().expand\_\;as(scores) \end{array}$$

Step 3 involves the computation of losses for image and text retrieval. During this stage, we evaluate the costs associated with image retrieval (cost_im) and text retrieval (cost_s). When determining these costs, various elements, such as diagonal scores, similarity scores, and a margin hyperparameter, are considered. The calculation process entails comparing the loss values to zero and utilizing the clamp function to prevent them from falling below zero. The specific calculation process is outlined in Equation (20).

(20) $$\begin{array}{c} cost\_s=\left(margin+scores-d1\right).clamp\left(min=0\right)\\ cost\;im=\left(margin+score-d2\right).clamp\left(\;min=0\right) \end{array}$$

Step 4 entails creating a mask matrix to nullify values positioned along diagonal within loss matrix. Since loss values on diagonal do not contribute to assessing the similarity between each sample and itself, this action is essential. Hence, they need to be adjusted to zero. The specific formula is given by Equation (21).

(21) $$\begin{array}{c} mask=torch.eye\left(scores.size\left(0\right)\right)>0.5\\ cost\_s=cost\_s.masked\_fill\_(mask,0)\\ cost\_im=cost\_im.maked\_fill\_\left(mask,0\right) \end{array}$$

Step 5: execute the ANS Strategy. In image retrieval, we identify the most challenging negative samples for each image (represented by each column) by selecting them based on the highest result of *(margin + scores − d*2*)*. The specific formula is given by Equation (22).

(22) $$\begin{array}{c} hard\_neg\_s=\left(margin+scores-d1\right).\max\left(1\right)\left[0\right]\\ hard\_neg\_im=\left(margin+scores-d2\right).\max\left(0\right)\left[0\right] \end{array}$$

Step 6: calculate the final trio loss by incorporating the deficits from text search, image search, and the ANS approach. We aggregate these losses and then deduct the cumulative scores of the adversarial negative samples from it to derive the ultimate loss value. The specific formula is given by Equation (23).

(23) $$\begin{array}{c} {triplet}_{loss}={cost}_{s}.sum()+{cost}_{im}.sum()\\ -hard\_neg\_s.sum()-hard\_neg\_im.sum() \end{array}$$

By employing the ANS strategy for the triplet loss, we can accelerate model training speed and enhance performance. This method assists in identifying the most formidable adverse instances related to tomato leaf disease, thus alleviating concerns regarding similarities among various ailments in tomato image-text retrieval across different modalities. As a result, it enhances the effectiveness of the model for retrieving images and text pertaining to conditions affecting tomato foliage.

**The FNE-ANS strategy is applied** exclusively during model training via triplet loss integration, however, it remains dormant during the model verification and application stages. During the model training process, we incorporate the proposed FNE-ANS methodology into the triplet dissimilarity measure to enhance the model’s performance. This strategy differs from existing methods [20,22,29,33,34] by excluding negative samples that are no longer exclusively hard negatives. Instead, we employ our FNE-ANS strategy, which comprehensively samples challenging instances while cutting false negatives. In the end, the arrangement of the triplet dissimilarity measure incorporating the FNE-ANS strategy is as illustrated below:

(24) $$\begin{array}{c} LANS={\left[margin-s(\bar{v},\bar{w})+s\left(\bar{v},{\bar{w}}_{fixANS}\right)\right]}_{+}\\ \;\;\;+{\left[margin-s(\bar{v},\bar{w})+s\left({\bar{v}}_{fixANS},\bar{w}\right)\right]}_{+}, \end{array}$$

(25) $$\;LFNE={\left[margin-s(\bar{v},\bar{w})+s\left(\bar{v},{\bar{w}}_{\mathrm{fixFNE}}\right)\right]}_{+}\;+{\left[margin-s(\bar{v},\bar{w})+s\left({\bar{v}}_{\mathrm{fixFNE}},\bar{w}\right)\right]}_{+,}$$

(26) Ltotal=alpha*LANS+(1−alpha)*LFNE

In the context of the triplet dissimilarity measure, we integrate the ANS loss component, which aims to penalize the model for misidentifying challenging negative samples. The margin serves as a benchmark for assessing the discernible disparity in the model’s scores between affirmative and adverse instances. $s(\overset{-}{v},\overset{-}{w})$ represents the forecasted rating of the system for a specific instance, where ${\overset{-}{w}}_{\mathrm{fixANS}}$ and ${\overset{-}{v}}_{\mathrm{fixANS}}$ denote predetermined embeddings for negative samples, determined by our ANS strategy, for text and images, respectively. Here, alpha serves as a weight parameter to adjust the contribution of the ANS loss within the overall loss function. This loss function is designed to impose a penalty on the model for incorrectly classifying instances as false negatives. The difference lies in the negative instances employed, such as prior methods.

Specifically, the symbols ${\overset{-}{w}}_{\mathrm{fixFNE}}$ and ${\overset{-}{v}}_{\mathrm{fixFNE}}$ represent the fixed negative embeddings derived from text and images through our FNE methodology. In this context, alpha is utilized to regulate the significance of the ANS loss within the comprehensive loss function.

The final total loss function forms two components: the ANS loss and the weighted sum of the triplet loss, with alpha regulating the weight distribution between them. During the training phase, we adjust the model’s parameters iteratively to minimize the total loss function, aiming to enhance the differentiation between positive and negative instances. Our emphasis falls particularly on addressing difficult negative samples and mitigating instances of false negatives. This contributes to refining the system’s efficacy and robustness.

During the evaluation and deployment of the model, we choose to prioritize similarity ranking over the FNE-ANS negative sample selection approach to enhance the effectiveness and precision of inference. Our approach adheres to a typical methodology, evaluating the similarity between the queried anchor and all specimens within the test collection, subsequently arranging them according to these resemblances. The highest-ranked instances, encompassing those in the top1, top5, and top10 positions, represent the outcomes of retrieval. These results are the instances that closely match the query anchor as per the model and are utilized in our integrated tomato image-text search project.

#### 2.2.4. AR

The AR method we propose primarily serves as a technique for generating adversarial samples. Adversarial training aids in preventing the model from overfitting to specific samples within the training set, thereby enhancing its ability to generalize to unseen data. Without leveraging AR, the model may lack adversarial robustness, meaning it inadequately accounts for perturbations introduced by adversarial samples during training. This susceptibility can lead the model to become overly sensitive to minor variations in the input space, consequently diminishing its overall robustness. Adversarial samples may be scarce within the dataset, or adversarial perturbations might not be adequately incorporated during sample generation. AR addresses this issue by introducing adversarial perturbations throughout the training process, thereby enriching the model’s learning of textual and visual features during training. This forces the model to better adapt to subtle variations in input, consequently bolstering its resistance to unknown perturbations and improving its resilience against adversarial attacks.

**Figure 6:**
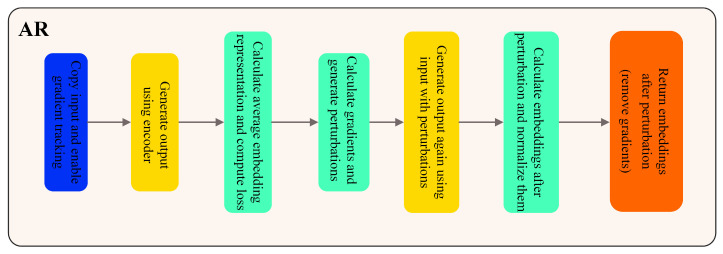
The schematic diagram of our proposed AR.

In Figure 6, AR first creates a gradient-enabled copy of the input tensor, ${\mathrm{inputs}}_{\mathrm{adv}}$, using *inputs.clone().requires_grad_(True)*. This ensures that gradients of the input can be computed during later backpropagation. Then, a conditional statement if ${\mathrm{attention}}_{\mathrm{mask}}$ is not none, it is used to check if an ${\mathrm{attention}}_{\mathrm{mask}}$ is provided. If so, the forward pass goes ahead as follows, according to the following:

(27) $$\mathrm{outputs}=\mathrm{encoder}\left({\mathrm{input}}_{\mathrm{ids}}={\mathrm{inputs}}_{\mathrm{adv}},\;{\mathrm{attention}}_{\mathrm{mask}}={\mathrm{attention}}_{\mathrm{mask}}\right)$$

Encode the model using ${\mathrm{input}}_{\mathrm{ids}}$ and the given ${\mathrm{attention}}_{\mathrm{mask}}$. Otherwise, if no attention mask is provided, encode using the following method:

(28) $$\begin{array}{c} outputs=encoder({\mathrm{pixel}}_{\mathrm{values}}=inputs_{\mathrm{adv}}) \end{array}$$

After obtaining the model’s output, we calculate the embedding representation of the adversarial sample through ${\mathrm{embeddings}}_{\mathrm{adv}}$ *= outputs.last_hidden_state.mean*(). This step uses the last hidden state of the model’s output and computes the text embedding by averaging across the sequence direction. Subsequently, the adversarial loss is computed using Equation (29). Here, ${\mathrm{embeddings}}_{\mathrm{adv}}$ is the embedding representation of the adversarial sample, embeddings denoting the embedding representation of the original sample, and $||\;|{|}_{2}^{2}$ denotes the L2 norm. The loss measures the difference between the embeddings of the adversarial sample and the original sample by computing the Euclidean distance between them, as shown in Equation (29):

(29) $$\mathrm{loss}=|\left|\mathrm{embedding}s_{\mathrm{adv}}\;-\mathrm{embeddings}\right|{|}_{2}^{2}$$

The objective of this stage is to measure the variance between the adversarial sample and the original sample within the embedding space, with the purpose of directing the adversarial sample towards proximity to the original sample during subsequent optimization.

Following this, we calculate the input’s loss gradient, as depicted in Equation (30):

(30) $$\begin{array}{c} grad={▽}_{{\mathrm{imputs}}_{\mathrm{adv}}}\left(\mathrm{loss}\right) \end{array}$$

Here, *grad* is the gradient of the adversarial *loss* with respect to the input. Subsequently, an element-wise sign function operation is applied to the gradient to generate the adversarial perturbation:

(31) $$\begin{array}{c} perturbations=\in *grad.sign \end{array}\left(\right)$$

The perturbations obtained in this step represent the direction and size of the perturbation. By adding this perturbation to the input copy, we update the input copy:

(32) $${\mathrm{input}}_{\mathrm{adv}}={\mathrm{input}}_{\mathrm{adv}}+\mathrm{perturbations}$$

We obtain the input copy, ${\mathrm{input}}_{\mathrm{adv}}$, which is subjected to adversarial perturbations while keeping the original features. This is done to encourage the model to learn more robust feature representations in an adversarial manner. In a context in which there are no gradient updates, we first choose different forward propagation approaches based on whether the attention mask is provided or not. If an attention mask is provided, we perform forward propagation using encoder (${\mathrm{input}}_{\mathrm{ids}}$ *=* ${\mathrm{input}}_{\mathrm{adv}}$, ${\mathrm{attention}}_{\mathrm{mask}}$ *=* ${\mathrm{attention}}_{\mathrm{mask}}$). If no attention mask is provided, we use encoder (${\mathrm{pixel}}_{\mathrm{values}}$ *=* ${\mathrm{input}}_{\mathrm{adv}}$) for forward propagation. This step ensures that the adversarial samples receive proper representations within the model. Next, we calculate the recalibrated embedding representation of the adversarial sample by averaging the last layer’s hidden states of the model output, as shown in Equation (33):

(33) $${\mathrm{embeddings}}_{\mathrm{adv}}={\mathrm{outputs}}_{\mathrm{adv}}.\mathrm{last}\_\mathrm{hidden}\_\mathrm{state}.\mathrm{mean}\left(1\right)$$

To further normalize the embedding representation of this adversarial sample, we employ L2 normalization using the following formula to ensure that the embeddings have unit length in Euclidean space, as shown in Equation (34):

(34) $$F.\mathrm{normalize}({\mathrm{embeddings}}_{\mathrm{adv}},\;p=2,\;\dim=-1)$$

Finally, by detaching the gradient using detach (), we successfully obtain the embedding representation of the adversarial sample. This adversarial sample representation has undergone re-calculation to capture adversarial loss and has been normalized to ensure it has unit length in Euclidean space. AR ensures that the adversarial sample keeps the semantic information of the input through regularization, providing effective guidance for the model’s learning and generalization.

The pseudo-code provided in Algorithm 2 outlines the AR algorithm.**Algorithm 2:** AR Algorithm**Input:** Tensor being the input data **Output:** Embeddings_adv: tensor of adversarial embeddings Pseudocode: def adv_perturbation (self, inputs, embeddings, encoder, attention_mask = None):     inputs_adv = inputs.clone().requires_grad_(True) # clone inputs tensor and set requires_grad to True     # Forward pass through the encoder      if attention_mask is not None:         outputs = encoder (input_ids = inputs_adv, attention_mask = attention_mask)      else:         outputs = encoder (pixel_values = inputs_adv)     # Compute adversarial loss based on embeddings    embeddings_adv = outputs.last_hidden_state.mean(1)    loss = embeddings_adv.sub (embeddings). norm (). sum ()     # Compute gradients and perturbations     grad = torch.autograd.grad (loss, inputs_adv, only_inputs = True)[0]     perturbations = self.epsilon * grad.sign()    inputs_adv = inputs_adv + perturbations    # Forward pass through the encoder with perturbed inputs    with torch.no_grad():        if attention_mask is not None:             outputs_adv = encoder (input_ids = inputs_adv, attention_mask = attention_mask)        else:            outputs_adv = encoder (pixel_values = inputs_adv)    # Compute adversarial embeddings and normalize    embeddings_adv = outputs_adv.last_hidden_state.mean(1)    embeddings_adv = F.normalize (embeddings_adv, p = 2, dim = −1)    return embeddings_adv.detach() # return detached adversarial embeddings

Through AR, there is an enrichment and enhancement of input feature sequences, resulting in a more thorough learning of features during model training. This directly leads to improvements in model retrieval performance. Additionally, it effectively addresses the problem of model overfitting, reduces reliance on training data, and enhances model robustness. In the training process of the LAFANet model, the proposed AR mechanism helps prevent model overfitting to training data, thereby enhancing its performance on unseen samples. Furthermore, AR strengthens the model’s resilience to adversarial perturbations and noise, making it more robust.

## 3. Experiments

### 3.1. Preparation of Experimental Environment

In this investigation, we ensured uniformity in hardware and software settings across all experiments to mitigate any potential impact of varying experimental conditions on LAFANet’s outcomes. The main training procedures were carried out with RTX A5000. LAFANet underwent training within the PyTorch 1.10.0 framework, operating under Cuda 11.3 and Python 3.8 environments.

The input images underwent uniform resizing to achieve dimensions of 256 × 256 pixels. Employing a dataset comprising 6000 pairs of image-text samples, we implemented five-fold cross-validation during the training phase. This approach allowed for a comprehensive evaluation of model performance while guarding against overfitting. Accordingly, the input data underwent a random split into proportions of 8:1:1. The training set served for model training, the validation set for appraising the trained model’s performance and parameter selection, and the test set for final performance assessment.

Throughout the training process, we used a batch size of 32 and a momentum memory module size of 8192. Training formed 80 epochs in total. We used the Adam optimizer, starting it with a learning rate of 0.00008.

We utilize BERT (bert-base-uncased) for extracting text features and ViT (vit-base-patch16) for extracting image features. In terms of the loss function, we utilized the proposed FNE-ANS approach to construct the triplet loss, with the margin value set to 0.2 to meet the specific task requirements during our model training. Moreover, we incorporated a tuning parameter denoted as α (as specified in Equation (16)) to regulate the sampling concentration, establishing it at 0.5. The selection of this parameter was made after extensive experimentation and fine-tuning to optimize performance efficiently.

### 3.2. Evaluation Metrics

In assessing the performance of LAFANet on the CRLDRD dataset, we adopted the methodology described in [35], employing *R@K* as a metric for evaluating retrieval outcomes. We comprehensively evaluated performance using three different *K* values: one, five, and ten. *R@K* describes the percentage of accurate retrievals made by the model among the top *K* entries within the ordered list.

For every inquiry, the system produces a ranked list, and R@K evaluates performance by counting the occurrences of the correct answer within the top K ranks. Its calculation is represented by the following Formula (35):

(35) $$\begin{array}{c} R@K=\frac{TP}{TP+FN} \end{array}$$

In the context of information retrieval, *TP* represents the count of accurately identified true answers among the top *K* selections, while *FN* signifies the count of true answers missed within the top *K* selections.

In the cross-domain image-text retrieval experiment concerning tomato leaf diseases, we assess model performance across various *K* values (*K* = 1, 5, 10). The importance of this approach lies in the fact that different values of *K* reflect various aspects of retrieval results in the tomato leaf disease task. Essentially, selecting the appropriate *K* values is vital for this task since various retrieval scenarios necessitate distinct results.

When *K =* 1, our priority lies in swiftly identifying the most pertinent outcome, an aspect of utmost importance in time-sensitive scenarios concerning tomato leaf disease detection. Nevertheless, at *K* = 10, it furnishes a wealthier array of data conducive to thorough investigations and analyses, including the extended surveillance of tomato leaf ailments.

Different *K* values can meet the needs of diverse usage contexts, guaranteeing that the model provides retrieval results suitable for agricultural use in various contexts.

### 3.3. Module Effectiveness Experiment

This segment furnishes an exhaustive account of the assessment criteria and parameters concerning LFA, FNE-ANS, and AR, demonstrating the efficacy of each inventive component within identical experimental conditions. The ensuing segment outlines the findings of the experiments.

#### 3.3.1. The Effectiveness of LFA

In this study, we implemented LFA to augment the image feature sequences derived from ViT and the text feature sequences obtained from BERT, with a comprehensive analysis of the experimental results provided in Table 1. We conducted an analysis that juxtaposed LFA against various self-attention mechanisms, encompassing Multi-Head Self-Attention, Stand-Alone Self-Attention, and Sparse Self-Attention. The results of our experiments indicate that LFA enhances the accuracy of image-text retrieval for tomato leaf diseases.

Our LFA demonstrates the most significant performance improvement when compared to Multi-Head Attention and Sparse Attention. This superiority stems from LFA’s capability to execute self-attention bidirectionally, employing two distinct weight calculation mechanisms. Subsequently, these mechanisms are amalgamated using adaptable fusion weights to effectively address various facets of input data. This enables the model to effectively manage the interference from the complex background of tomato leaves, thereby improving retrieval accuracy. The experimental results illustrating LFA’s performance advantage in tasks involving the retrieval of images and text related to tomato leaf diseases are presented in Table 2.

#### 3.3.2. Effectiveness of FNE-ANS

**Figure 7:**
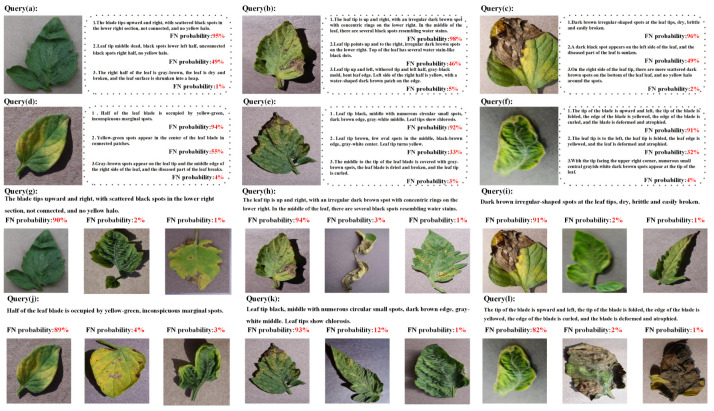
Verification instances illustrating the rates of false negatives (FN) concerning negative samples during the application of the FNE-ANS tactic.

In this study, we propose the FNE-ANS methodology, which emphasizes the selection of adverse instances for utilization in the triplet loss computation. The efficacy of this approach is illustrated in Figure 7, which primarily aims to validate the FNE-ANS methodology’s ability to assess the probability of incorrect negatives among the selected negative samples. We assessed the instances of incorrect negatives to confirm that the approach we employed, incorporating ANS, identifies challenging negative instances and effectively reduces false negatives through FNE. Consequently, this results in the selection of more impactful negative samples for model training within the triplet loss framework.

Figure 7 presents several examples of false negative tests, with image anchors illustrated at the top and text anchors at the bottom. Each example has a false negative rate for negative samples defined in our dataset. We see that certain negative samples share significant semantic information with their respective anchors and should be matched with them. In earlier studies, these negative samples with high similarity to anchors were treated as challenging negative samples and were encouraged during the application of the FNE strategy, leading to conflicts between optimization aims.

With our FNE-ANS strategy, depicted in the diagram, we assess the likelihood of adverse instances being false negatives, and afterward employ a weighted sampling scheme to diminish their presence in triplets. Additionally, we note that samples with low false negative probabilities show distinct semantic information from the anchors. In brief, our FNE-ANS approach effectively gauges the likelihood of false negatives among negative samples, tackling the complexities of adverse samples and facilitating the detection of a wide range of adverse instances, all while mitigating inter-class similarity issues across different tomato leaf disease categories. This method demonstrates novelty in categorizing diseases, leading to enhanced performance in retrieving images and text related to tomato leaf diseases.

#### 3.3.3. Effectiveness of AR

**Figure 8:**
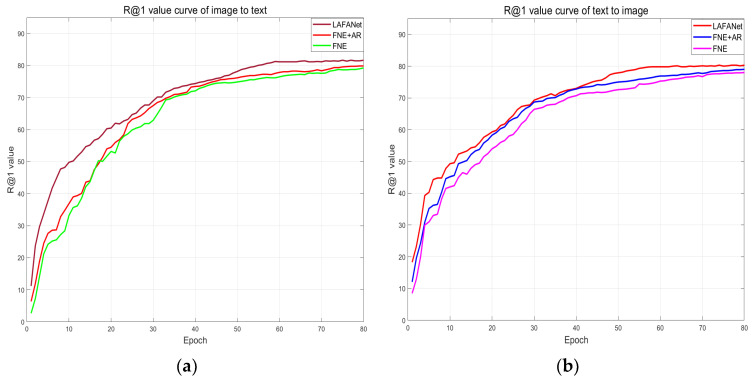
The training R@1 value trend curve. (**a**) Text-to-image retrieval R@1 curve; (**b**) image-to-text retrieval R@1 curve.

In this paper, we propose AR to apply regularization perturbations to the extracted features of the tomato leaf disease cross-modal image-text retrieval model LAFANet and visualize the effects of the AR algorithm on enriching the extracted features through regularization perturbations in Figure 8. Drawing inspiration from regularization principles, we propose the regularization perturbation AR algorithm to enrich the extracted image and text features. We reflect the effectiveness of the regularization perturbations on our model training through intuitive changes in image-text retrieval accuracy during training.

To explore the effectiveness of the AR algorithm, we visualize and compare the changes in retrieval accuracy during training of our model LAFANet, the baseline model FNE+AR, and FNE. In Figure 8, the experimental results confirm the rationale behind our proposed AR algorithm and prove its superiority. The AR algorithm proves its effectiveness in improving the retrieval accuracy during model training in Figure 8.

From Figure 8, in the first training epochs, the accuracy improvement of FNE+AR is faster than that of FNE due to the enriching effect of the AR algorithm on the extracted features. During the training process, the curves of FNE + AR and LAFANet are smoother compared to FNE alone, showing that the AR algorithm enhances the stability of model training. In the later epochs of training, both FNE + AR and LAFANet achieve higher accuracy than FNE. LAFANet, with the added support of our other innovations, notably outperforms FNE, while FNE + AR, with only the inclusion of the AR algorithm, also proves a visible but slight improvement over FNE.

In conclusion, our proposed AR algorithm demonstrates its effectiveness in enhancing the retrieval performance and training stability of LAFANet by augmenting the extracted features of the model through regularization perturbation, thus affirming its efficiency for matching images and textual descriptions related to tomato leaf diseases across different modalities.

### 3.4. Ablation Study

We conducted extensive controlled experiments on the cross-modal image-text retrieval dataset for tomato leaf disease to evaluate the effectiveness of each innovative part in our approach. The results are presented in Table 3 (all results are based on a single model). The findings led to the following observations:

Solely using the FNE baseline model but simultaneously removing LFA, ANS, and AR resulted in a significant performance drop, validating the efficacy of the proposed modules, such as LFA, ANS, and AR, in bolstering model performance.

Removing solely ANS led to a marginal decrease in performance. This is because ANS broadens the range of adverse instances while heightening the difficulty of identifying such instances. By integrating ANS with the original FNE approach of the model, more demanding negative samples capable of mitigating false negatives were generated. The application of these samples for sampling provided more comprehensive and valuable training of negative samples.

When exclusively excluding LFA, there was a further decrease in performance. This emphasizes the significant contribution of LFA to improving image and text attributes, resulting in enhanced semantic harmony between images and text and ultimately enhancing the performance of image-text retrieval.

Similarly, when removing AR, there was a slight decrease in model performance. This suggests that our AR enriches the input training data through regularization perturbations, leading to a more comprehensive learning of image-text features by the model, thus achieving better performance.

To enhance the precision and dependability of the model, we utilized K-fold cross-validation throughout the training phase. This method involves multiple iterations of dataset allocation during training, where a part of the data is used for training and the remaining part is used for validation. By considering various data scenarios comprehensively, this approach ensures the generalization performance of the model.

We implemented a quintuple cross-validation technique, apportioning 80% of the training dataset and 10% of the validation dataset for assessing the model’s performance, while the remaining 10% was held out for testing purposes. This allowed us to showcase the different effects of LAFANet and the standard FNE model in the task of matching images with text descriptions related to tomato leaf disease.

**Figure 9:**
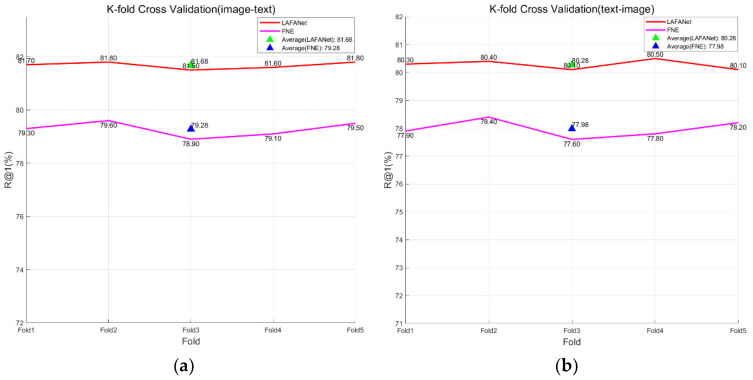
K-fold cross validation. (**a**) Results of image retrieval for text; (**b**) results of text retrieval for image.

In this study, we conducted five experiments, recording the accuracy of each experiment, and calculated the average accuracy to obtain the result, as shown in Figure 9. The findings indicate that our LAFANet consistently surpassed the pre-enhanced model FNE across randomly partitioned training and validation sets. This indicates that LAFANet performed well across different validation sets, improving its ability to adapt, and confirming its reliability and effectiveness in matching images with text descriptions related to issues affecting tomato leaves.

### 3.5. Performance Comparison

To evaluate the effectiveness of our proposed LAFANet model, we compared it with several existing techniques and baseline methods on the designated tomato leaf disease image-text retrieval dataset CRLDRD, as outlined in Table 4. Various fundamental techniques are utilized, including visual–semantic embedding strategies (like VSE∞ [21], CAMERA [30], our basic model FNE [25], and SAEM [39], alongside others), along with methods focusing on attention (such as SCAN [22], AOQ [40], IMRAM [41], DIME [23], NAAF [29], and more). Visual–semantic embedding methods usually have better retrieval efficiency during inference, but may face performance limitations due to insufficient cross-modal interactions. Conversely, attention-focused methods, which explore fine-grained cross-modal interactions, can achieve superior performance, although they might need higher computational resources.

However, the advent of pre-trained models like BERT [30] has led to a gradual reduction in the performance gap between these two approaches. Our baseline model FNE leverages pre-trained models, resulting in leading performance.

The recent technique VSE∞ is indeed a form of visual–semantic embedding. It is worth noting that many methods further enhance performance by integrating multiple models. We conducted a fair comparison and introduced a composite model that aggregates similarity scores from two models trained with distinct random.

In Table 4, we present the quantitative performance comparison results between LAFANet and other ultramodern techniques. In comparison to baseline model FNE, LAFANet exhibits significant improvements in R@1, R@5, and R@10. For image retrieval to text, it achieves increases of 2.4%, 2.3%, and 2.7%, respectively; for text retrieval to images, the enhancements are 2.3%, 0.7%, and 0.6%, respectively. Among the methods of CA and VSE, NAAF and VSE∞ stand out as top performers compared to other baseline methods. NAAF, an extension of SCAN, enhances the similarity calculation by prioritizing mismatched segment similarity, integrating positive and negative effects in attention computation, resulting in improved performance for the CA method. VSE∞ boosts performance through the opportunistic exploration of challenging negative instances, underscoring the significance of acquiring difficult negative samples for aligning images with text.

Our LAFANet tackles the challenges associated with negative samples, including false positives, by employing the innovative FNE-ANS method for comprehensive negative sample selection, surpassing all current technical approaches in evaluation metrics. LAFANet outperforms VSE∞ by 4.6% and 3.0% in R@1 for both retrieval directions, and compared to NAAF, LAFANet improves R@1 by 4.3% and 3.2% in both directions. This proves the effectiveness of FNE-ANS, which comprehensively samples negative samples, preserves their challenge, and cuts false positives. The bidirectional hybrid self-attention mechanism, known as LFA, elevates the performance of retrieval by amplifying both image and text features. AR further enriches the features extracted by the model through regularization perturbations, leading to more comprehensive training and improved retrieval accuracy. These enhancements ultimately lead to improved performance in cross-modal image-text retrieval for tomato datasets.

## 4. Discussion

The main contributions of this study to addressing multiple issues in tomato leaf disease image-text retrieval can be summarized by four aspects. 1. To address the difficulties presented by intricate backgrounds and indistinct subject borders in image retrieval, we utilized ViT and BERT models for extracting detailed feature sequences from both image and text inputs, thereby incorporating textual information. Subsequently, we proposed LFA, a mechanism that selectively enhances feature sequences of images and text, further exploring their significant semantic information. The innovation of LFA enables the model to capture key semantic information more accurately in both images and text, thereby exhibiting excellent performance in disease retrieval tasks. This approach effectively reduces retrieval inaccuracies caused by complex backgrounds or blurred subject edges in image-text pair retrievals. 2. To address potential detection errors arising from intra-class variability of tomato leaf diseases, we focused our research on six highly prevalent and pathogenic tomato diseases. We meticulously curated and processed the dataset, selecting representative images of diseases. Based on this, we created the Cross-Modal Tomato Leaf Disease Image-Text Retrieval Dataset (TLDITRD), focusing on six common tomato leaf diseases. This dataset includes comprehensive textual descriptions, establishing strong connections between images and related text. The creation of the TLDITRD dataset plays a crucial role in our research, providing us with a perfect model validation object. 3. To address the challenge of limited correlation between images and text caused by the diverse and numerous textual descriptions during the retrieval process, we conducted extensive exploration into the semantic associations among different data types. By combining adversarial negative sample selection strategy with false negative elimination strategy, we proposed the FNE-ANS method. We integrated this method into a triplet loss function, imposing strong constraints on the model. Through the implementation of this approach, the model’s resilience and capacity for generalization are notably enhanced, showcasing exceptional performance in practical settings and demonstrating heightened adaptability. Furthermore, this approach substantially boosts the precision of text data extraction, leading to notable enhancements in matching retrieval accuracy. This research outcome offers unparalleled assistance for prompt disease prevention and precise diagnosis in crop management, marking a significant advancement in precision agriculture. To address the problem of limited input data and feature diversity in improving model performance during the model training process, we proposed AR. Adversarial training enables the model to better adapt to changes in different environments and data distributions, reducing sensitivity to input variations, and enhancing the model’s robustness in practical application scenarios. Additionally, AR not only improves the model’s robustness but also enhances its defense capabilities, which is particularly important in fields such as crop disease detection, where various noises, environmental changes, and attacks may occur in practical applications, making the model more suitable for complex and dynamic real-world scenarios. Introducing this optimization strategy into model training has yielded meaningful results in improving model performance and providing valuable insights for further research.

Although our model, LAFANet, achieved the best performance on the TLDITRD dataset in comparative experiments, outperforming other state-of-the-art models with retrieval accuracies of 81.7% for image-to-text retrieval and 80.3% for text-to-image retrieval at R1, this suggests that further enhancement of model effectiveness is possible. The results indicate that, while our model accurately matches images with text in most cases, occasional misclassifications may still occur in rare abnormal situations, possibly due to different stages or evolution of leaf diseases. This aspect requires further research and improvement to enhance the robustness and accuracy of the model. Although these misclassifications do not affect disease recognition, they may interfere with our model’s temporal assessment of leaf disease stages, potentially affecting the timely prevention and management of tomato leaf diseases.

Addressing the current limitations of the model, in the future we plan to resolve the issue of temporal assessment retrieval of leaf disease stages by conducting fine-grained temporal grading retrieval. This aims to further improve the model’s granularity retrieval accuracy and robustness during different stages of leaf disease development. Fine-grained temporal grading retrieval can establish a comprehensive early warning system by determining the severity of leaf diseases, enabling accurate retrieval of images and text related to tomato leaf diseases. Through this system, we can assist agricultural workers in early detection of tomato leaf diseases, facilitating timely measures to mitigate the impact and reduce losses caused by leaf diseases. Additionally, in the initial stages of diseases, it may be challenging to determine whether leaves are affected by diseases based solely on image and text information. Future considerations include the development of a sophisticated cross-modal retrieval application framework by integrating Internet of Things (IoT) sensor technology with image-text retrieval methods. The framework would be utilized for fine-grained disease transmission retrieval processes.

## 5. Conclusions

This paper focuses on crop disease issues in agriculture. Exploring the identification of crop leaf ailments, we present LAFANet, a cross-modal framework for retrieving images and text, for swift and precise retrieval of diverse insights on tomato leaf diseases, facilitating timely and accurate preventive measures. The contributions of this work are summarized as follows. (1) We developed the TLDITRD dataset, featuring six typical images of tomato leaf diseases along with corresponding textual descriptions, to pioneer the integration of cross-modal retrieval within tomato leaf disease research. (2) To mitigate retrieval inaccuracies stemming from intricate backgrounds, we propose LFA to further enhance sequences of image and text characteristics, extracting significant semantic data from both modalities. (3) To investigate semantic associations across diverse modalities, we introduce the FNE-ANS approach aimed at identifying adversarial negative instances within the triplet function. This method aims to mitigate false negatives and impose constraints on the model, thereby enhancing its performance. (4) To enhance model generalization and accuracy, we propose using AR to train the model with adversarial perturbations, improving the model’s robustness to minor variations in input data and obtaining a more robust model. (5) Extensive experiments on the TLDITRD dataset confirm that our proposed LAFANet achieves accuracies of 81.7%, 83.3%, and 90.0% for image-to-text retrieval and 80.3%, 93.7%, and 96.3% for text-to-image retrieval, respectively. LAFANet surpasses the baseline models for FNE, advanced VSE∞, and NAAF techniques, boosting R@1 accuracy by 2.4%, 4.6%, and 4.3%, respectively, for image-to-text retrieval, and by 2.3%, 3.0%, and 3.2% for text-to-image retrieval tasks. Experimental analyses demonstrate that components like LFA, ANS, and AR effectively contribute to the enhancement of model performance. Specifically, (a) in the images to text task, introducing LFA improves R@1 by 0.9%, ANS by 0.5%, and AR by 0.6% compared to the baseline model FNE. (b) In the text to images task, introducing LFA improves R@1 by 1.1%, ANS by 0.9%, and AR by 1.0% compared to the baseline model FNE. The integration of LFA, ANS, and AR modules substantially boosts image-text retrieval effectiveness, leading to enhanced accuracy and recall, consequently facilitating finer semantic correlation between images and text.

Although advancements have been achieved in research, lingering concerns remain, notably pertaining to determining the seriousness of leaf diseases to establish a comprehensive early warning system for precise retrieval of tomato leaf disease images and texts. The formation of leaf diseases is closely related to the parasitic process of bacteria, covering multiple stages from local to global. Although different diseases show unique characteristics, the same disease may present differently in different environments. By detailing the characteristics of different disease stages, the severity of the disease can be further decided. This not only aids in the early detection of diseases but also facilitates prompt actions to mitigate the disease’s impact, thus preventing losses in production and unnecessary depletion of resources. Moreover, during the initial stages of a disease, it can be challenging to determine if leaves are affected based solely on visual inspection and textual information. Future avenues for research involve the comprehensive integration of Internet of Things (IoT) sensor technology with image-text search methods to develop a sophisticated cross-modal retrieval framework for detecting disease transmission processes. This fusion strives to enrich disease early warning systems by providing a finer-grained understanding of the interplay between images and textual data.

## Figures and Tables

**Table 1:** Mainstream image-text retrieval methods.

Methods of Literature	Contribution
Frome et al. [19]	Employing CNN and Skip-Gram for feature extraction from both visual and textual inputs.
VSE++ [20]	Strengthening CNN and Skip-Gram with an online hard negative selection technique in the triplet loss function, enabling deeper investigations.
Chen et al. [21]	A universal pooling technique, which dynamically decides the best pooling strategy through learning distinct weights for each dimension of ranking.
SCAN [22]	Selectively integrates image regions and textual elements to gauge the similarity between images and text.
Wei et al. [12]	A method incorporating both cross-attention and self-attention mechanisms to effectively capture interactions within and across modalities.
Qu et al. [23]	A dynamic routing mechanism for model embedding, allowing the dynamic selection of embedding paths according to input.
NAAF [24]	Extends the SCAN methodology, aiming to refine similarity computation by mitigating the drawbacks associated with mismatched fragments.
FNE [25]	A novel approach to address false negatives by employing sampling techniques on negative instances.

**Table 2:** Assessment of the efficiency of the LFA component on the test set.

Methods	Images to Text			Text to Images		
	R@1 (%)	R@5 (%)	R@10 (%)	R@1 (%)	R@5 (%)	R@10 (%)
FNE [25]	79.3	81.0	87.3	78.0	93.0	95.7
FNE + Multi-Head Self-Attention [36]	79.6	81.5	87.9	78.4	93.1	95.7
FNE + Stand-Alone Self-Attention [37]	80.1	81.8	88.3	78.9	93.2	95.9
FNE + Sparse Self-Attention [38]	79.7	81.6	88.1	78.6	93.1	95.8
Ours (FNE + LFA)	80.2	82.1	88.7	79.1	93.3	96.0

**Table 3:** The quantitative results of ablation experiments.

Methods	Images to Text			Text to Images		
	R@1 (%)	R@5 (%)	R@10 (%)	R@1 (%)	R@5 (%)	R@10 (%)
FNE [25]	79.3	81.0	87.3	78.0	93.0	95.7
FNE + LFA	80.2	82.1	88.7	79.1	93.3	96.0
FNE + ANS	79.8	81.7	88.3	78.9	93.1	95.9
FNE + AR	79.9	82.0	88.5	79.0	93.2	95.9
FNE + LFA + ANS	80.9	82.5	89.2	79.4	93.3	96.1
FNE + LFA + AR	81.2	82.8	89.4	79.6	93.5	96.2
FNE + ANS + AR	80.7	82.2	89.0	79.3	93.2	96.1
Ours	81.7	83.3	90.0	80.3	93.7	96.3

**Table 4:** Tomato leaf disease text-image retrieval test set retrieval results.

Method	Images to Text			Text to Images		
	R@1 (%)	R@5 (%)	R@10 (%)	R@1 (%)	R@5 (%)	R@10 (%)
SCAN [22]	72.1	75.2	84.3	71.3	89.2	91.8
SAEM [39]	73.0	75.8	84.7	72.2	89.7	92.6
VSRN [42]	74.1	76.6	85.2	73.7	90.6	93.8
IMRAM [41]	75.4	77.3	85.7	75.2	91.2	94.3
GSMN [43]	76.4	78.5	86.4	76.0	91.9	94.8
AOQ [40]	76.7	78.9	86.8	76.5	92.3	95.1
CAMERA [30]	76.6	78.8	86.7	76.2	92.1	94.9
DIME [23]	76.9	79.1	87.0	77.6	92.8	95.5
VSE∞ [21]	77.1	78.9	86.9	77.3	92.6	95.3
NAAF [29]	77.4	79.3	87.1	77.1	92.5	95.3
FNE [25]	79.3	81.0	87.3	78.0	93.0	95.7
Ours (LAFANet)	81.7	83.3	90.0	80.3	93.7	96.3

## Data Availability

The datasets in this study are available upon request from the corresponding author.
